# Association between subclinical right ventricular alterations and aerobic exercise capacity in type 2 diabetes

**DOI:** 10.1016/j.jocmr.2024.101120

**Published:** 2024-10-28

**Authors:** Abhishek Dattani, Jian L. Yeo, Emer M. Brady, Alice Cowley, Anna-Marie Marsh, Manjit Sian, Joanna M. Bilak, Matthew P.M. Graham-Brown, Anvesha Singh, Jayanth R. Arnold, David Adlam, Thomas Yates, Gerry P. McCann, Gaurav S. Gulsin

**Affiliations:** a Department of Cardiovascular Sciences, University of Leicester, and the National Institute for Health and Care Research Leicester Biomedical Research Centre, Leicester, UK; b Diabetes Research Centre, College of Life Sciences, University of Leicester, Leicester, UK

**Keywords:** Type 2 diabetes, Right ventricle, Exercise capacity, Cardiovascular imaging, Heart failure

## Abstract

**Background:**

Type 2 diabetes (T2D) leads to cardiovascular remodeling, and heart failure has emerged as a major complication of T2D. There is a limited understanding of the impact of T2D on the right heart. This study aimed to assess subclinical right heart alterations and their contribution to aerobic exercise capacity (peak oxygen consumption; peak VO_2_) in adults with T2D.

**Methods:**

Single center, prospective, case-control comparison of adults with and without T2D, and no prevalent cardiac disease. Comprehensive evaluation of the left and right heart was performed using transthoracic echocardiography and stress cardiovascular magnetic resonance. Cardiopulmonary exercise testing on a bicycle ergometer with expired gas analysis was performed to determine peak VO_2_. Between group comparison was adjusted for age, sex, race, and body mass index using analysis of covariance (ANCOVA). Multivariable linear regression, including key clinical and left heart variables, was undertaken in people with T2D to identify independent associations between measures of right ventricular (RV) structure and function with peak VO_2_.

**Results:**

Three hundred and forty people with T2D (median age 64 years, 62% (211) male, mean glycated hemoglobin (HbA1c) 7.3%) and 66 controls (median age 58 years, 58% (38) male, mean HbA1c 5.5%) were included. T2D participants had markedly lower peak VO_2_ (adjusted mean 20.3 (95% confidence interval (CI): 19.8–20.9) vs 23.3(22.2–24.5) mL/kg/min, P < 0.001) than controls and had smaller left ventricular (LV) volumes and LV concentric remodeling. Those with T2D had smaller RV volumes (indexed RV end-diastolic volume: 84 (82–86) vs 100 (96–104) mL/m, P < 0.001) with evidence of hyperdynamic RV systolic function (global longitudinal strain (GLS): 26.3 (25.8–26.8) vs 23.5 (22.5–24.5)%, P < 0.001) and impaired RV relaxation (longitudinal peak early diastolic strain rate (PEDSR): 0.77 (0.74–0.80) vs 0.92 (0.85–1.00) s^-1^, P < 0.001). Multivariable linear regression demonstrated that RV end-diastolic volume (β = −0.342, P = 0.004) and RV cardiac output (β = 0.296, P = 0.001), but not LV parameters, were independent determinants of peak VO_2_.

**Conclusion:**

In T2D, markers of RV remodeling are associated with aerobic exercise capacity, independent of left heart alterations.

## Graphical abstract

**Figure:**
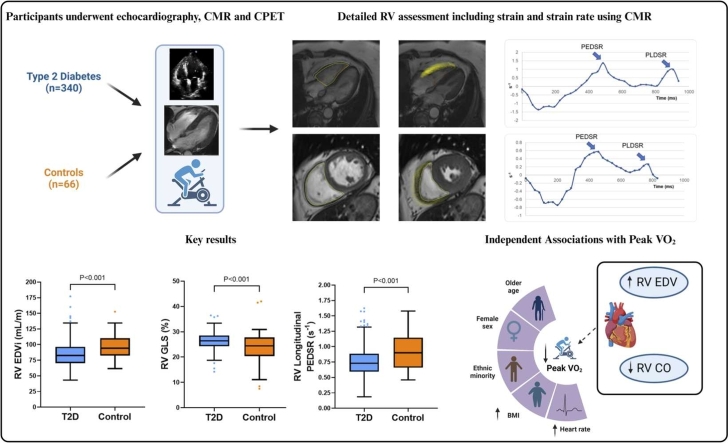

## 1 Introduction

Type 2 diabetes (T2D) is a multisystem disease with rapidly increasing global prevalence [1]. Numerous studies have shown that T2D is associated with several subclinical cardiac alterations that precede the development of symptomatic heart failure (HF), which has emerged as a major complication of T2D [2], [3]. Most evidence for cardiovascular remodeling in T2D has been focused on the left heart with T2D being associated with smaller left ventricle (LV) volumes, concentric LV remodeling, and reduced LV global longitudinal strain (GLS) [3]. There is, however, a paucity of data relating to the impact of T2D on the right heart.

Right ventricle (RV) dysfunction can occur in the absence of overt LV alterations, and is associated with exercise intolerance, poor functional capacity, and worse outcomes irrespective of the underlying mechanism [4]. Given the systemic biological mechanisms involved in T2D and the intimate relationship between the LV and RV, it would be surprising for T2D not to have a detrimental impact on the RV.

Previous work on the impact of T2D on the RV has largely assessed patients using transthoracic echocardiography [5], [6]. However, transthoracic echocardiography has inherent limitations such as dependence on acoustic windows, which is particularly relevant in people with T2D, and the complex shape of the RV significantly restricts its visualization and accurate quantification of volumes using echocardiography [7].

Cardiovascular magnetic resonance (CMR) imaging is the reference standard technique for the quantification of RV volumes and mass [8] and tissue tracking enables the quantification of myocardial strain and strain rates for detailed assessment of RV systolic and diastolic mechanics [9]. The use of CMR in the assessment of the RV in T2D has so far been limited to relatively small studies with no assessment of functional impact [10], [11].

Numerous studies have demonstrated reduced exercise capacity in people with T2D, both in the presence [12], [13] and absence of cardiac disease [14], [15], [16]. While this has been linked to impaired skeletal muscle physiology, the role cardiac structure and function have in exercise capacity is not fully understood. Cardiopulmonary exercise testing (CPET) is a direct, objective, gold-standard method for quantification of aerobic exercise capacity, peak oxygen consumption (peak VO_2_) [17], and has been shown to predict cardiovascular events including death [18], [19] and the development of HF [20], [21]. To our knowledge, no studies have assessed the impact of CMR-derived RV parameters on exercise capacity in people with T2D.

We aimed to: (1) compare subclinical alterations in right heart structure and function in a multi-ethnic cohort of people with T2D in comparison to non-T2D controls, and (2) assess whether features of subclinical right heart disease are independently associated with peak VO_2_ in those with T2D.

## 2 Research design and methods

### 2.1 Study design and participants

This was a prospective, cross-sectional observational study (Prevalence and Determinants of Subclinical Cardiovascular Dysfunction in Adults with Type 2 Diabetes Mellitus; NCT03132129). Participants were recruited from primary care services in Leicestershire, UK, with support from the NIHR East Midlands Clinical Research Network. Participants were aged 18–75 years with a diagnosis of T2D and no prior history, signs, or symptoms of cardiovascular disease (including symptomatic coronary, peripheral or cerebrovascular disease, valvular heart disease, arrhythmias, or HF). Exclusion criteria were diagnosis of type 1 diabetes, estimated glomerular filtration rate (eGFR) <30 mL/min/1.73 m^2^, or absolute contraindication to CMR. A group of controls aged 18–75 without T2D or a history of cardiovascular disease was also enrolled for comparison (NCT03132129, ISRCTN42661582). Ethical approval was provided by the UK Health Research Authority Research Ethics Committee (17/WM/0192, 14/EM/0056). All participants provided written informed consent.

### 2.2 General assessments

Demographics, medical history, and anthropometric measurements were collected. A fasting blood sample was collected for biochemical profiling including renal function, lipid profile, HbA1c, high-sensitivity troponin I, and natriuretic peptides. Samples were collected on the same day as the imaging procedures and analyzed in an accredited National Health Service pathology lab at the University Hospitals of Leicester, UK. Ambulatory blood pressure was measured over 24 hours with a validated blood pressure monitor (Space lab model 90207, Snoqualmie, Washington) [22].

Activity levels were measured over 7 days using a GENEActiv accelerometer (ActivInsights Ltd., Cambridgeshire, UK) with monitors set to record accelerations at 100 Hz. Activity levels are presented as time in moderate-vigorous physical activity (MVPA) bouts lasting 1–5 minutes per day with detailed methodology reported elsewhere [23].

### 2.3 Transthoracic echocardiography

Transthoracic echocardiography was performed by one of two accredited operators using an iE33b system with an X5-1 transducer (Phillips Medical Systems, Best, Netherlands). Images were acquired and reported as per the American Society of Echocardiography guidelines.

### 2.4 Cardiopulmonary exercise testing

Exercise capacity was assessed in a temperature-controlled room using an incremental symptom-limited CPET (CASE Exercise Testing System, General Electric Healthcare, Chicago, Illinois) with a bicycle ergometer (eBike Comfort, General Electric Healthcare, Chicago, Illinois). Calibration was performed prior to each assessment. A 1-minute ramp protocol was used with workload increments calculated based on participant age, sex, height, and weight [24]. Gas analysis was performed using a Ganshorn Powercube and appropriate post-processing software (Ganshorn LF8) using a 30-second rolling mean of breath-by-breath data. Peak VO_2_ was determined as the highest value and indexed to weight for analysis. Participants who did not achieve a respiratory exchange ratio (RER) of ≥1.0 were excluded from further CPET analysis to mitigate the confounding effects of tests in which reaching peak VO_2_ was highly unlikely.

### 2.5 Cardiovascular magnetic resonance

A 3 T scanner (Siemens Skyra or Vida, Siemens Healthineers, Erlangen, Germany) was used to perform a stress CMR scan as per a standardized protocol as previously described [25]. In brief, both long- and short-axis cine imaging were undertaken using a balanced steady-state free precession technique with coverage of the whole heart. Perfusion imaging was performed at rest and during pharmacological stress using 140–210 µg/kg/min of adenosine infused for 3–5 minutes. Stress and rest perfusion images were obtained at basal, mid-ventricular, and apical levels following the administration of a gadolinium-based contrast agent (0.075 mmol/kg Gadoteric acid, Dotarem, Guerbet, Villepinte, Paris France). Quantitative myocardial blood flow analyses were performed using a dual-sequence gradient echo method with inline automated reconstruction and post-processing for myocardial blood flow quantification at basal, mid-ventricular, and apical slice positions [26]. A pre- and post-contrast T1 map was obtained at basal and mid-ventricular level using a modified inversion recovery Look-Locker technique.

### 2.6 Image analysis

All CMR images were batch-analyzed offline by a single observer (A.D.) with blinding to participant details using cvi42 (Version 5.13, Circle Cardiovascular Imaging, Calgary, Alberta, Canada). A subset of participants (n = 15) underwent repeat RV contouring by an expert in the field (G.P.M.). LV and left atrial (LA) image analysis were performed in a similar fashion as previously described using automated contouring with adjustments made for clear and obvious errors [25]. RV volumes and mass were quantified by manually contouring the endocardial and epicardial borders at the end-diastolic and end-systolic phases in the short-axis slices using the built-in automated contouring tool with manual correction. Right atrial area was calculated in the four-chamber view using the automated tool to contour throughout the cardiac cycle. Parameters were indexed to height where appropriate.

**Fig. 1:**
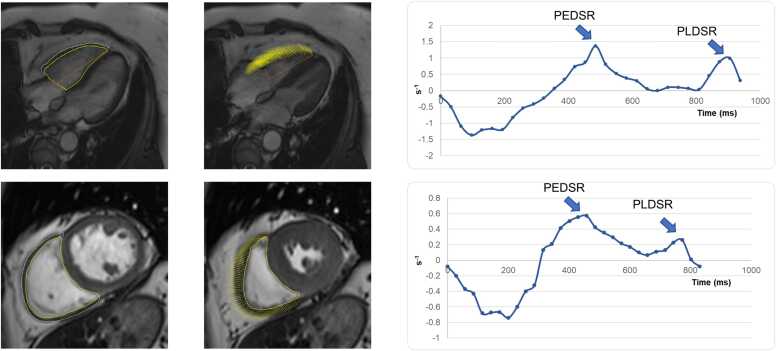
Assessment of RV strain using CMR. RV endocardial and epicardial contours were drawn in the long axis (top) and short axis views (bottom) in end-diastole (left) and end-systole (middle). Beyond volumetric, mass, and systolic function assessment, curves were generated for diastolic strain rate for the longitudinal (top right) and circumferential direction (bottom right). *CMR* cardiovascular magnetic resonance, *PEDSR* peak early diastolic strain rate, *PLDSR* peak late diastolic strain rate, *RV* right ventricular

Tissue tracking was used to quantify LV and RV strain. LV strain indices were calculated as previously described [25]. RV myocardial strain was quantified by contouring the endocardial and epicardial borders of the RV slices in the short-axes and four-chamber view at end-diastole, providing curves for systolic and diastolic strain and strain rate (Fig. 1). The quality of tracking and strain curves were visually assessed prior to data extraction and participants with poor tracking or curves were excluded from further analysis. Subsequently, curves were analyzed to provide the following key strain parameters: GLS, global circumferential strain (GCS), longitudinal PEDSR, circumferential PEDSR, longitudinal peak late diastolic strain rate (PLDSR), and circumferential PLDSR. GLS and GCS are presented as absolute values such that lower values indicate worse myocardial mechanics [27]. Given differences in heart rate between participants, strain rate was normalized to a nominal heart rate of 60 beats per minute using the following equation: (strain rate/heart rate) * 60. The main pulmonary artery diameter was measured as per clinical standards using Half-Fourier Acquisition Single-shot Turbo spin Echo imaging.

### 2.7 Statistical analysis

Baseline characteristics were compared between groups using an independent T-test, Mann–Whitney U test, or Chi-squared test as appropriate. Imaging and CPET variables were compared between groups using either ANCOVA or binary logistic regression, with covariates including age, sex, race, and body mass index, and data are presented as adjusted mean (95% confidence interval). Confirmation of differences between key variables was assessed using regression models in the whole cohort with additional adjustment for systolic blood pressure, smoking history, and activity levels. Correlations of imaging variables with peak VO_2_ were first assessed using Pearson correlation coefficient in people with T2D and controls separately, and also assessed for collinearity. In the T2D group, in order to identify independent associations of peak VO_2_, those variables which were found to significantly correlate with peak VO_2_ were then entered into a multivariable linear regression model together with key clinical variables (age, sex, race, body mass index, smoking status, systolic blood pressure, heart rate, eGFR, and HbA1c) and LV imaging variables (E/e’ and myocardial perfusion reserve) known to be linked with exercise capacity [25]. Statistical analysis was performed using SPSS Statistics (version 28.0, IBM Corp., New York, New York USA). A P < 0.05 was considered statistically significant.

## 3 Results

### 3.1 Baseline characteristics

**Fig. 2:**
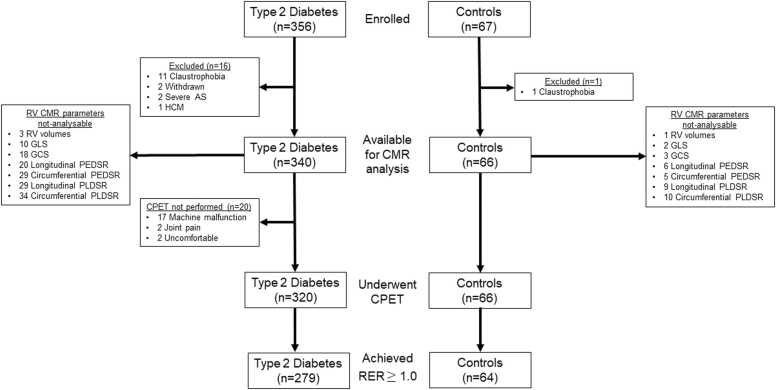
Study flow diagram. Summary of study enrollment and exclusions. *AS* aortic stenosis, *HCM* hypertrophic cardiomyopathy, *RV* right ventricle, *GLS* global longitudinal strain, *GCS* global circumferential strain, *PEDSR* peak early diastolic strain rate; *PLDSR* peak late diastolic strain rate, *CPET* cardiopulmonary exercise testing, *RER* respiratory exchange ratio, *CMR* cardiovascular magnetic resonance

Following exclusions (Fig. 2), a total of 340 people with T2D and 66 controls were included in this analysis. Table 1 describes the key baseline characteristics of the two groups. The groups were well matched for sex and race distribution but the T2D group was older, had a higher body mass index, heart rate, and HbA1c level, with a higher prevalence of hypertension and hyperlipidemia in comparison to the control group. Accordingly, more subjects in the T2D group were on anti-hypertensive and lipid-lowering medications, and had lower cholesterol but higher triglyceride in comparison to the control group. There was no significant difference in activity levels between the two groups.

**Table 1:** Baseline characteristics.

	T2D (n = 340)	Controls (n = 66)
Age (y)	64 (58–69) *	58 (54–65)
Male sex	211 (62%)	38 (58%)
*Race*		
White	259 (76%)	53 (80%)
Asian	70 (21%)	12 (18%)
Other/mixed	11 (3%)	1 (2%)
BMI (kg/m^2^)	29 (26–33) *	26 (23–30)
Systolic BP (mmHg)	127 ± 12 *	123 ± 12
Diastolic BP (mmHg)	74 ± 7	75 ± 8
Heart rate (bpm)	76 ± 13 *	64 ± 9
MVPA (min)	10.5 (5.7–17.1)	11.2 (6.2–20.8)
*Medical history*		
Diabetes duration (y)	9 (5–14)	-
Hypertension	196 (58%) *	10 (15%)
Hyperlipidemia	237 (70%) *	10 (15%)
Obstructive sleep apnea	15 (4%)	1 (2%)
*Smoking*		
Never	189 (55%)	38 (58%)
Ex-smoker	123 (36%)	26 (39%)
Current	29 (9%)	2 (3%)
*Medications*		
ACEi/ARB	164 (48%) *	6 (9%)
Alpha blocker	19 (6%) *	0
Beta blocker	24 (7%)	1 (2%)
CCB	89 (26%) *	5 (8%)
Diuretic	31 (9%)	1 (2%)
Aspirin	10 (3%)	0
Statin	238 (71%) *	10 (15%)
Fibrate	9 (3%)	0
Insulin	47 (14%) *	0
Thiazolidinediones	3 (1%)	0
Sulphonylurea	50 (15%) *	0
Metformin	241 (71%) *	0
GLP-1 agonist	29 (9%) *	0
DPP-4 inhibitor	51 (15%) *	0
SGLT2 inhibitor	71 (21%) *	0
*Biochemistry*		
HbA1c (%)	7.3 ± 1.2 *	5.5 ± 0.4
HbA1c (mmol/mol)	56 ± 13 *	36 ± 4
HOMA-IR	5.6 (3.4–10.1) *	1.6 (1.1–3.0)
eGFR (mL/min/1.73 m^2^)	85 ± 15	84 ± 11
Total cholesterol (mmol/L)	4.2 (3.6–4.9) *	5.7 (4.8–6.0)
HDL (mmol/L)	1.3 (1.1–1.6) *	1.6 (1.3–2.2)
LDL (mmol/L)	2.1 (1.6–2.7) *	3.2 (2.5–3.8)
Triglyceride (mmol/L)	1.5 (1.1–2.0) *	1.1 (0.9–1.4)
NT-proBNP (ng/L)	45 (0–78)	48 (0–135)
hsTnI (ng/L)	2.5 (2.4–4.4)	2.5 (2.0–4.0)
Urine ACR <3 mg/mmol 3–30 mg/mmol >30 mg/mmol	200 (73%) 64 (23%) 9 (3%)	30 (79%) 8 (21%) 0

Data are presented as numbers (%) of cases, means ± standard deviation, or medians (interquartile range) as appropriate.

*T2D* type 2 diabetes, *BMI* body mass index, *BP* blood pressure, *ACEi* angiotensin converting enzyme inhibitor, *ARB* angiotensin II receptor blocker, *CCB* calcium channel blocker, *GLP-1* glucagon-like peptide-1, *DPP-4* dipeptidyl peptidase 4, *SGLT2* sodium-glucose co-transporter-2, *HOMA-IR* homeostatic model assessment for insulin resistance, *HDL* high-density lipoprotein, *LDL* low-density lipoprotein, *NT-proBNP* N-terminal prohormone of brain natriuretic peptide, *hsTnI* high-sensitivity troponin I, *ACR* albumin:creatinine ratio, *MVPA* moderate-vigorous physical activity, *eGFR* estimated glomerular filtration rate. *P < 0.05 compared to control group.

### 3.2 Imaging data

Table 2 summarizes imaging data for the two groups. CMR demonstrated that the T2D group had smaller LV volumes with minimal difference in LV mass resulting in a higher LV mass:volume ratio. Although LV systolic strain parameters were similar, there was a lower longitudinal and circumferential LV PEDSR in the T2D group compared to the controls. The T2D group had a higher extracellular volume fraction compared to the control group and lower myocardial perfusion reserve. There was no difference in extracellular volume fraction, stress myocardial blood flow, or myocardial perfusion reserve in T2D patients with and without LGE (Supplementary Table S1).

**Table 2:** Imaging analysis in people with type 2 diabetes and controls.

	n	T2D (n = 340)	n	Controls (n = 66)	P value*
*LV CMR data*					
LV EDV (mL)	336	129 (126–131)	65	151 (144–157)	**<0.001**
LV EDV index (mL/m)	336	76 (74–77)	65	88 (84–91)	**<0.001**
LV ESV index (mL/m)	336	26 (25,26)	65	30 (28–32)	**<0.001**
LV EF (%)	336	67 (66,67)	65	66 (65–68)	0.689
LV cardiac index (L/min/m)	336	3.8 (3.7–3.9)	65	3.8 (3.6–4.0)	0.953
LV mass (g)	336	114 (111–116)	65	124 (119–129)	**<0.001**
LV mass index (g/m)	336	67 (66–68)	65	72 (69–75)	**0.001**
LV mass:volume (g/mL)	336	0.90 (0.89–0.92)	65	0.82 (0.79–0.86)	**<0.001**
LV GLS (%)	335	16.2 (16.0–16.5)	66	16.7 (16.2–17.3)	0.109
LV GCS (%)	335	19.5 (19.2–19.7)	65	19.4 (18.8–20.0)	0.919
LV longitudinal PEDSR (s^−1^)†	329	0.54 (0.52–0.55)	64	0.61 (0.56–0.65)	**0.004**
LV circumferential PEDSR (s^−1^)†	323	0.71 (0.69–0.74)	63	0.87 (0.81–0.92)	**<0.001**
LV longitudinal PLDSR (s^−1^)†	326	0.66 (0.64–0.68)	64	0.66 (0.61––70)	0.969
LV circumferential PLDSR (s^−1^)†	323	0.64 (0.62–0.66)	63	0.65 (0.61–0.69)	0.604
Rest MBF (mL/g/min)	306	0.66 (0.64–0.68)	64	0.68 (0.64–0.73)	0.413
Stress MBF (mL/g/min)	304	1.80 (1.74–1.87)	65	2.07 (1.93–2.21)	**0.001**
Myocardial perfusion reserve	297	2.83 (2.73–2.92)	64	3.12 (2.91–3.33)	**0.015**
Late gadolinium enhancement	335	72 (22%)	65	8 (12%)	0.537
Extracellular volume fraction (%)	325	27.4 (27.0–27.7)	44	25.8 (24.8–26.9)	**0.007**
*RV CMR data*					
RV EDV (mL)	337	143 (139–146)	65	172 (164–179)	**<0.001**
RV EDV index (mL/m)	337	84 (82–86)	65	100 (96–104)	**<0.001**
RV EDV/LV EDV ratio	335	1.12 (1.10–1.13)	65	1.14 (1.11–1.18)	0.112
RV ESV index (mL/m)	337	38 (37–39)	65	46 (43–48)	**<0.001**
RV EF (%)	337	55 (54,55)	65	54 (53–56)	0.775
RV cardiac index (L/min/m)	337	2.9 (2.9–3.0)	65	3.0 (2.9–3.2)	0.375
RV mass index (g/m)	332	17.4 (17.1–17.7)	64	19.4 (18.6–20.2)	**<0.001**
RV mass:volume (g/mL)	332	0.21 (0.21–0.22)	64	0.19 (0.19–0.21)	**0.002**
RV GLS (%)	330	26.3 (25.8–26.8)	64	23.5 (22.5–24.5)	**<0.001**
RV GCS (%)	322	16.0 (15.6–16.3)	63	14.8 (14.0–15.6)	**0.010**
RV longitudinal PEDSR (s^−1^)†	320	0.77 (0.74–0.80)	60	0.92 (0.85–1.00)	**<0.001**
RV circumferential PEDSR (s^−1^)†	311	0.56 (0.54–0.57)	61	0.60 (0.56–0.64)	0.075
RV longitudinal PLDSR (s^−1^)†	311	1.23 (1.18–1.27)	57	1.13 (1.02–1.24)	0.118
RV circumferential PLDSR (s^−1^)†	306	0.32 (0.31–0.34)	56	0.26 (0.22–0.30)	**0.005**
*Atrial CMR data*					
LA maximum volume index (mL/m)	330	35 (34–36)	64	42 (39–45)	**<0.001**
LA EF (%)	328	62 (61–63)	64	58 (56–61)	**0.007**
RA maximum area index (cm^2^/m)	328	12 (12)	64	15 (14,15)	**<0.001**
RA EF (%)	328	56 (55–57)	64	51 (49–54)	**0.001**
*Pulmonary CMR data*					
PA diameter (mm)	334	24.2 (23.9–24.6)	63	23.7 (22.9–24.6)	0.316
PA diameter/height (mm/m)	334	14.4 (14.1–14.6)	63	13.9 (13.4–14.4)	0.142
*Echocardiography data*					
E wave	335	70 (69–72)	46	70 (66–74)	0.956
A wave	337	81 (80–83)	46	76 (72–81)	**0.049**
E/A ratio	335	0.88 (0.86–0.90)	46	0.95 (0.89–1.00)	**0.031**
E/e’	332	9.1 (8.9–9.4)	46	8.9 (8.3–9.6)	0.562
MV deceleration time	332	229 (223–234)	46	246 (230–262)	**0.045**
Tricuspid regurgitation present	340	74 (22%)	46	18 (39%)	**0.005**
Diastolic function grading Normal Grade I Indeterminate Grade II/III		62 (19%) 239 (74%) 22 (7%) 1 (0%)		14 (33%) 25 (58%) 4 (9%) 0	0.167

Continuous data are presented as adjusted mean (95% confidence interval). Values indexed to height where appropriate.

*T2D* type 2 diabetes, *RV* right ventricle, *LV* left ventricle, *RA* right atrium, *LA* left atrium, *PA* pulmonary artery, *EDV* end-diastolic volume, *ESV* end-diastolic volume, *EF* ejection fraction, *GLS* global longitudinal strain, *GCS* global circumferential strain, *PEDSR* peak early diastolic strain rate, *PLDSR* peak late diastolic strain rate, *MBF* myocardial blood flow, *MV* mitral valve, *CMR* cardiovascular magnetic resonance

* Adjusted for age, sex, race, and body mass index

† Strain rates normalized to heart rate using formula: (strain rate/heart rate) × 60

People with T2D also had smaller RV volumes compared to controls (indexed RV end-diastolic volume (EDVi): 84 (82–86) vs 100 (96–104) mL/m, P < 0.001). The T2D group had lower RV mass and had higher RV mass:volume ratio (0.21 (0.21–0.22) vs 0.19 (0.19–0.21) g/mL, P = 0.002) in comparison to the control group. Although RV EF was similar between the groups (55 (54, 55) vs 54 (53–56) %, P = 0.775), both RV GLS (26.3 (25.8–26.8) vs 23.5 (22.5–24.5) %, P < 0.001) and RV GCS (16.0 (15.6–16.3) vs 14.8 (14.0–15.6) %, P = 0.010) were higher in the T2D group compared to controls. RV longitudinal PEDSR was lower (0.77 (0.74–0.80) vs 0.92 (0.85–1.00) s^-1^, P < 0.001), but circumferential PLDSR was higher (0.32 (0.31–0.34) vs 0.26 (0.22–0.30) s^-1^, P = 0.005) in the T2D group. Regression models (Supplementary Tables S2 and S3) including the whole cohort showed that T2D was associated with lower RV volumes and RV mass index as well as higher RV systolic strain and lower RV longitudinal PEDSR.

Left and right atrial volumes were lower in the T2D group with higher left and right atrial EF compared to the controls.

On echocardiography, E/A ratio and A wave were lower in the T2D group, although both groups had similar E/e’ values. There was no significant difference in LV diastolic function grades between the two groups as per American Society of Echocardiography guidelines, with the majority of participants having either normal diastolic function or grade I diastolic dysfunction.

Thirteen control participants had HbA1c levels that fell in the prediabetes range (5.7–6.4%) and therefore a sensitivity analysis was performed excluding these participants (Supplementary Table S4), which demonstrated consistent findings.

CMR RV analysis of a subset of participants performed by a second observer demonstrated minimal inter-observer variability (Supplementary Table S5).

### 3.3 CPET data

A total of 320 participants with T2D and all 66 controls underwent CPET (Fig. 2). Of these, 279 participants with T2D and 64 controls achieved an RER ≥1.0 and were included in further analyses. Exercise duration was similar between the two groups, but the T2D group had significantly lower peak VO_2_ (20.3 (19.8–20.9) vs 23.3 (22.2–24.5) mL/kg/min, P < 0.001) and peak workload (123 (119–127) vs 161 (153–170) watts, P < 0.001) compared to the control group (Supplementary Table S6).

### 3.4 Associations with peak VO_2_

Upon correlation assessment within the T2D group (Supplementary Table S7), RV EDV, end-systolic volume (ESV), cardiac output, and RV EDV/LV EDV ratio had a positive association with peak VO_2_, and RV circumferential PLDSR was inversely associated with peak VO_2_. Of the LV parameters, only LV cardiac output was associated with peak VO_2_, as was LA EF and RA maximum area. RV EDV and ESV were significantly associated with each other (R = 0.881, P < 0.001) and therefore RV ESV was excluded from further association analyses. RV cardiac output and LV cardiac output were also significantly associated (R = 0.790, P < 0.001) but since RV cardiac output had a stronger association with peak VO_2_, only RV cardiac output was entered into the final regression model.

Multivariable linear regression (Table 3) demonstrated that RV EDV and RV cardiac output were independently associated with peak VO_2_ along with age, sex, race, body mass index, and heart rate (R = 0.731, R^2^ = 0.534, P < 0.001). Post hoc power calculations for this model showed 99.3% power (effect size 0.15, α 0.05, sample size 279, and 17 variables). The addition of imaging parameters improved the strength of the regression model in comparison to a model only made up of clinical variables (R = 0.665, R^2^ = 0.442; Supplementary Table S8). The same multivariable regression performed on the control group demonstrated that RV EDV was the only RV parameter to be independently associated with peak VO_2_ (Supplementary Table S9).

**Table 3:** Multivariable linear regression model for predicting weight-adjusted peak VO_2_ in the type 2 diabetes group who achieved respiratory exchange ratio ≥1.0.

	Unstandardized coefficients		Standardized coefficients	P value
	B	Standard error	Beta	
Age	−0.266	0.051	−0.351	**<0.001**
Male sex	4.202	0.721	0.378	**<0.001**
Non-White	−2.459	0.609	−0.235	**<0.001**
Body mass index	−0.441	0.070	−0.396	**<0.001**
Smoking status	−0.347	0.452	−0.044	0.444
Systolic BP	−0.041	0.025	−0.094	0.112
Heart rate	−0.112	0.037	−0.206	**0.003**
eGFR	−0.015	0.021	−0.043	0.468
HbA1c %	−0.261	0.264	−0.057	0.323
E/e’	0.016	0.136	0.007	0.907
Myocardial perfusion reserve	−0.020	0.359	−0.003	0.955
LA EF	−0.003	0.035	−0.005	0.936
RV EDV	−0.050	0.017	−0.342	**0.004**
RV cardiac output	1.097	0.312	0.296	**0.001**
RV circumferential PLDSR	−2.167	1.540	−0.088	0.161
RV EDV/LV EDV ratio	2.979	2.152	0.081	0.168
RA maximum area	0.159	0.087	0.135	0.069

R = 0.731, R square = 0.534, Adjusted R square = 0.490, P < 0.001

*RER* respiratory exchange ratio, *BP* blood pressure, *LA* left atrial, *EF* ejection fraction, *RV* right ventricle, *EDV* end-diastolic volume, *PLDSR* peak late diastolic strain rate, *LV* left ventricle, *RA* right atrium, *eGFR* estimated glomerular filtration rate.

## 4 Discussion

This is the first study to assess the impact of subclinical RV alterations on exercise capacity in asymptomatic adults with T2D using the combined gold-standard techniques of CMR and CPET. Our main findings were that subclinical alterations in structure and function are evident in both the left and right heart in T2D with significant reductions in RV volumes and evidence of hyperdynamic RV systolic function but impaired RV relaxation. However, markers of RV remodeling, and not LV parameters, are independently associated with exercise capacity in people with T2D. This suggests that RV alterations play a central role in the downstream development of exercise intolerance in this group and a greater emphasis may need to be placed on right heart assessment during risk stratification of people with T2D than current guidelines dictate.

Several studies have attempted to characterize RV alterations in T2D, predominantly using transthoracic echocardiography. These studies have provided conflicting data with some demonstrating no difference in RV size [28], [29] compared to controls, while others have shown smaller RV in people with T2D [6]. Some of these conflicting data may be related to lack of indexing for RV parameters in the former studies compared to the latter study which indexed to body surface area and it is well recognized that indexing variables are important when comparing groups [30]. Furthermore, echocardiographic strain analysis has demonstrated reduced diastolic strain rate but has also shown reduced systolic strain which is contradictory to our study [6]. There are significant limitations with RV assessment during transthoracic echocardiography related to the restricted views possible making accurate assessment of volumes difficult [7]. Additionally, the thin wall of the RV complicates strain analysis with circumferential strain being particularly difficult to assess using echocardiography, and there is poor agreement between echocardiographic assessment of RV deformation and CMR-measured tissue tracking techniques [31].

Few studies have used CMR to assess RV alterations in people with T2D and, to our knowledge, none have gone on to assess the associations with exercise capacity. In keeping with our findings, Widya et al. [10] showed smaller RV volumes compared to controls using CMR and demonstrated evidence of RV diastolic dysfunction using transthoracic echocardiography. This study was in a smaller group of patients (n = 78 with T2D) and was only performed in males. In a larger study (n = 143 with diabetes) incorporating data from the UK Biobank, Jensen et al. confirmed smaller RV volumes [32], but neither of these studies assessed RV strain or exercise capacity. One feasibility study performed RV strain analysis using CMR in people with T2D [11] who showed, contrary to our findings, reduced GLS and GCS. Their strain values, however, were outside recognized physiological values suggesting differences in analytical techniques. Furthermore, their cohort was smaller (n = 104 with T2D), younger and had a significantly lower BMI compared to our cohort.

There is a plethora of data showing morphological and functional alterations in the left heart of people with T2D including smaller LV and LA volumes, LV concentric remodeling, and both systolic and diastolic dysfunction [3]. From our data, similarities can be seen in the right compared to the left heart, such as smaller RV and right atrial volumes, RV concentric remodeling, and alterations in RV diastolic strain rate patterns. Despite similar RV EF, we saw that this group of asymptomatic people with T2D had hyperdynamic RV systolic strain when compared to controls which is not in keeping with LV changes often seen. Moreover, E/e’ was not raised in our cohort of T2D suggesting the RV changes are not simply a result of changes within the LV.

Several mechanisms may explain the changes in RV remodeling and function observed in our cohort, primarily relating to cardiopulmonary interactions. Our understanding of the role of the cardiopulmonary axis in HF with preserved ejection fraction (HFpEF) is growing. Increases in pulmonary artery pressure occur as a result of elevated LA filling pressures in HFpEF, but these patients often also develop an increase in pulmonary vascular resistance leading to significant morbidity and mortality [33]. T2D is a systemic disease affecting both the systemic and pulmonary circulation, with T2D being associated with pulmonary hypertension independent of coronary artery disease, hypertension, HF, or smoking [34]. Pre-clinical studies have suggested pulmonary artery endothelial dysfunction may occur in T2D via the induction of NADPH oxidase [35]. Our T2D group had larger mean pulmonary artery diameter compared to controls (although did not reach statistical significance) and did not have significant pulmonary hypertension (lack of symptoms or significant tricuspid regurgitation) which may indicate a subclinical increase in pulmonary vascular resistance, which may explain some of the changes found in our study. For example, an increased RV afterload could lead to a compensatory increase in systolic function (as evidenced by systolic strain) resulting from an intrinsic increase in contractile function [36]. Alternatively, this could be a result of catecholamine-induced inotropy linked to the effects of leptin on the sympathetic nervous system which can be seen in some phenotypes of obesity-related HF [37]. Although increased LV afterload in the acute setting can lead to increased LV volume as a result of the Frank-Starling mechanism, differences in RV anatomy mean that this plays a smaller role in RV adaptation [38]. Furthermore, the systemic nature of T2D [3] may have a direct impact on structural changes in the RV in a similar fashion to the LV and thus may explain why our T2D cohort had smaller volumes. As with the LV, the biological mechanisms behind smaller RV volumes in T2D require further work.

The key novelty of our data is the first demonstration of the impact of these RV alterations on exercise capacity in this cohort of patients, with RV EDV being inversely associated with peak VO_2_, and RV cardiac output being directly associated with peak VO_2_. In people with T2D, cardiorespiratory fitness is an independent predictor of incident HF [21] and peak VO_2_ provides important prognostic information in patients with HF [39]. Our group has previously demonstrated that LV markers of dysfunction such as E/e’ and myocardial perfusion reserve are important determinants of exercise capacity in similar cohort of patients, [25], [40]. Other groups have shown that the LV basal segmental diastolic velocity, as measured by echocardiography, is an independent predictor of exercise capacity together with age, sex, BMI, and HbA1c [40]. However, these studies did not account for the crucial role played by the right heart. Strikingly, in this study, we have shown that RV, but not LV, markers of remodeling were the main cardiac determinants of peak VO_2_. Two relatively small studies have shown the importance of RV parameters for the determination of exercise capacity but these were performed in patients with chronic systolic HF [41], [42]. To our knowledge, no studies have demonstrated this finding in patients with HFpEF or in at-risk populations such as people with T2D. The biological link between the RV changes and exercise capacity could be related to the inherent functions of the cardiopulmonary axis and alterations in RV-pulmonary arterial coupling but this would require further mechanistic research with invasive measurements. Studies directly investigating RV function during exercise may also reveal the importance of subclinical RV changes in increasing RV cardiac output during exercise.

People with T2D are at high risk of developing HF (stage A HF) [43]. Importantly, in people with T2D, each additional feature of stage B HF leads to an incremental increase in risk of events [44], thus highlighting the importance of accurate categorization of patients. Current guidelines have little focus on the RV changes seen in stage B HF and although thresholds for the definition of stage B HF for LV parameters have been suggested, none exist for the RV [43]. Given the implication of RV alterations in this group of patients, more research is needed for our understanding of the role the right heart plays in the prognosis of patients at early stages of HF.

## 5. Limitations

Key strengths of this study are its prospective design and large sample size for the nature of this study, with good representation of females and ethnic minority groups representative of our regional population. We used detailed cardiac phenotyping using gold-standard imaging techniques for RV analysis, together with CPET for quantification of exercise capacity which represents major strengths of the study. The rigorous exclusion of patients with cardiovascular disease or those who had sub-optimal effort during CPET are further strengths of the data.

Limitations include a small difference in age between groups, though this was corrected for in statistical analysis and by performing the regression model separately in the groups. The control group is relatively small in comparison to the T2D group as the main aim of this study was to assess the impact of RV changes in the T2D group. The sample size and power calculations were therefore based on the number of T2D patients needed for an appropriately powered regression model. The regression model in the control group is presented for information in the supplementary, but is likely to be underpowered. This study is a cross-sectional observational study without long-term follow-up for HF outcomes, but we did use peak VO_2_ which is a strong surrogate measure of cardiovascular and HF outcomes in T2D. As with all multiple regression models, there is a risk of unknown or omitted variables which may result in over-estimation in the size of variance attributed to variables within the model. In order to minimize this risk, however, we did perform correlations for all imaging variables with peak VO_2_ and added all key clinical variables into this model. There are discrepancies between the RV and LV cardiac index which are likely a result of different techniques used for RV and LV contours (manual vs automated). Furthermore, the RV volumes were generated using the LV short-axis stack rather than a dedicated RV axial stack which may have resulted in the exclusion of the right ventricular outflow tract in some T2D and control participants. Given the nature of the study, we did not perform invasive measurement of right heart or pulmonary artery pressures and therefore we are unable to confirm the impact on pulmonary vascular resistance.

## 6. Conclusions

In conclusion, in this prospective study of asymptomatic people with T2D, we have demonstrated smaller RV volumes with hyperdynamic RV systolic function alongside impaired RV diastolic function. RV EDV and RV cardiac output are independently associated with exercise capacity in people with T2D. Whether these changes are seen in the evolution of chronic RV decompensation in T2D requires longitudinal studies, and serial imaging would be needed in order to evaluate the natural history of diabetic cardiomyopathy.

Funding

A.D. received funding from the British Heart Foundation through a Clinical Research Training Fellowship (FS/CRTF/20/24069). J.L.Y., E.M.B., and G.P.M. received funding from the National Institute for Health and Care Research (NIHR) United Kingdom through a Research Professorship award (RP-2017-08-ST2-007). G.S.G. received funding from the British Heart Foundation through a Clinical Research Training Fellowship (FS/16/47/32190) and Travel Fellowship (FS/TF/21/33008). DA was supported by the 10.13039/501100000274British Heart Foundation (BHF) PG/13/96/30608, the NIHR rare disease translational collaboration and BeatSCAD. DA has received research funding from Abbott vascular to support a clinical research fellow. He has also received funding from AstraZeneca Inc. for unrelated research and has undertaken consultancy for General Electric Inc. to support research funds. He receives royalties from Elsevier Inc.for the ECG made Practical and ECG Problems books and holds the following unrelated patents (EP3277337A1, PCT/GB2017/050877, UK patent application number 2211616.4). We acknowledge support from the NIHR Leicester Biomedical Research Centre and the NIHR Leicester Clinical Research Facility.

## Author contributions

Thomas Yates: Writing—review and editing. Gaurav S. Gulsin: Writing—review and editing, Supervision, Methodology, Investigation. Emer M. Brady: Writing—review and editing, Methodology. Gerry P. McCann: Writing—review and editing, Supervision, Resources, Methodology, Funding acquisition, Conceptualization. Jian L. Yeo: Supervision, Methodology, Investigation. Anna-Marie Marsh: Investigation. Alice Cowley: Writing—review and editing, Supervision, Project administration. Joanna M. Bilak: Investigation. Manjit Sian: Investigation. Anvesha Singh: Writing—review and editing. Matthew P. M. Graham-Brown: Writing—review and editing. David Adlam: Supervision, Resources, Methodology, Investigation. Jayanth R. Arnold: Writing—review and editing. Abhishek Dattani: Writing—original draft, Project administration, Methodology, Investigation, Formal analysis, Data curation, Conceptualization

## Ethics approval and consent

Ethical approval was provided by the UK Health Research Authority Research Ethics Committee (17/WM/0192). All participants provided written informed consent.

Consent for publication

Not applicable.

## Declaration of competing interests

The authors declare that they have no known competing financial interests or personal relationships that could have appeared to influence the work reported in this paper.

## Data Availability

The dataset used during the current study is available from the corresponding author on reasonable request.
