# Supplementary material for: Association between subclinical right ventricular alterations and aerobic exercise capacity in type 2 diabetes
Source: J Cardiovasc Magn Reson. 2024 Oct 28;26(2):101120. doi: 10.1016/j.jocmr.2024.101120 (PMC11663768; doi:10.1016/j.jocmr.2024.101120)
Supplement: Supplementary file 1 — Supplementary material [file mmc1.docx]

**Supplementary Table S1: Comparison of extracellular volume fraction and perfusion in type 2 diabetes patients with and without late gadolinium enhancement**

|  | LGE present  (n=72) | LGE absent  (n=268) | P value |
| --- | --- | --- | --- |
| ECV (%) | 27.4±3.7 | 26.9±2.7 | 0.271 |
| Stress MBF | 1.74±0.62 | 1.80±0.54 | 0.466 |
| MPR | 2.91±0.87 | 2.80±0.83 | 0.372 |

Abbreviations: ECV = extracellular volume fraction, LGE = late gadolinium enhancement, MBF = myocardial blood flow, MPR = myocardial perfusion reserve.

**Supplementary Table S2: Multivariable regression across the whole cohort to demonstrate impact of type 2 diabetes on key right ventricular volumes and mass parameters**

|  | RV EDVi | | RV ESVi | | RV mass index | |
| --- | --- | --- | --- | --- | --- | --- |
|  | Standardized Beta | P value | Standardized Beta | P value | Standardized Beta | P value |
| Age | -0.097 | **0.049** | -0.114 | **0.020** | -0.042 | 0.389 |
| Male sex | 0.402 | **<0.001** | 0.500 | **<0.001** | 0.358 | **<0.001** |
| Non-white | -0.228 | **<0.001** | -0.177 | **<0.001** | -0.247 | **<0.001** |
| Body mass index | 0.334 | **<0.001** | 0.293 | **<0.001** | 0.425 | **<0.001** |
| Smoking status | 0.031 | 0.518 | 0.057 | 0.227 | 0.068 | 0.147 |
| Systolic BP | 0.105 | **0.029** | 0.019 | 0.684 | 0.045 | 0.343 |
| MVPA | -0.024 | 0.598 | -0.031 | 0.498 | -0.095 | **0.037** |
| Type 2 diabetes | -0.278 | **<0.001** | -0.220 | **<0.001** | -0.202 | **<0.001** |

Abbreviations: RV = right ventricle, EDVi = indexed end-diastolic volume, ESVi – indexed end-systolic volume, BP = blood pressure, MVPA = moderate-vigorous physical activity

|  | RV GLS | | RV GCS | | RV Longitudinal PEDSR | | RV Circumferential PLDSR | |
| --- | --- | --- | --- | --- | --- | --- | --- | --- |
|  | Standardized Beta | P value | Standardized Beta | P value | Standardized Beta | P value | Standardized Beta | P value |
| Age | 0.089 | 0.133 | 0.166 | **0.007** | -0.061 | 0.329 | 0.307 | **<0.001** |
| Male sex | -0.137 | **0.015** | -0.122 | **0.038** | -0.043 | 0.458 | -0.122 | **0.036** |
| Non-white | -0.135 | **0.018** | -0.103 | 0.082 | -0.033 | 0.578 | 0.083 | 0.164 |
| Body mass index | -0.004 | 0.942 | -0.055 | 0.360 | -0.117 | 0.054 | 0.130 | **0.032** |
| Smoking history | -0.054 | 0.345 | -0.037 | 0.534 | -0.031 | 0.604 | 0.011 | 0.849 |
| Systolic BP | 0.007 | 0.904 | 0.082 | 0.174 | 0.062 | 0.309 | -0.072 | 0.235 |
| MVPA | 0.125 | **0.026** | 0.051 | 0.376 | 0.067 | 0.254 | -0.044 | 0.449 |
| Type 2 diabetes | 0.257 | **<0.001** | 0.154 | **0.011** | -0.186 | **0.002** | 0.135 | **0.025** |

**Supplementary Table S3: Multivariable regression across the whole cohort to demonstrate impact of type 2 diabetes on key right ventricular strain parameters**

Abbreviations: RV = right ventricle, GLS = global longitudinal strain, GCS = global circumferential strain, PEDSR = peak early diastolic strain rate, PLDSR = peak late diastolic strain rate, BP = blood pressure.

**Supplementary Table S4: Sensitivity analysis excluding controls with pre-diabetes**

|  | T2D  (n=340) | Controls  (n=53) |
| --- | --- | --- |
| Age (years) | 64 (58-69) * | 56 (53-65) |
| Male sex | 211 (62%) | 28 (53%) |
| BMI (kg/m^2^) | 29 (26-33) * | 26 (23-29) |
| Systolic BP (mmHg) | 127±12* | 122±14 |
| Heart rate (bpm) | 76±13 * | 69±7 |
| Hypertension | 196 (58%) * | 7 (13%) |
| Hyperlipidaemia | 237 (70%) * | 7 (13%) |
| HbA1c (%) | 7.3±1.2 * | 5.3±0.3 |
| eGFR (mL/min/1.73m^2^) | 85±15 | 82±10 |
| Total cholesterol (mmol/L) | 4.2 (3.6-4.9) * | 5.7 (4.6-6.1) |
| CMR | | |
| RV EDVi | 84 (82-86) ^†^ | 101 (96-106) |
| RV ESVi | 38 (37-39) ^†^ | 46 (43-49) |
| RV ejection fraction | 55 (54-55) | 55 (52-56) |
| RV cardiac index | 2.9 (2.9-3.0) | 3.1 (2.9-3.3) |
| RV mass index | 17.4 (17.1-17.7) ^†^ | 19.7 (18.8-20.6) |
| RV mass:volume | 0.21 (0.21-0.22) ^†^ | 0.20 (0.19-0.21) |
| RV GLS | 26.3 (25.9-26.7) ^†^ | 22.9 (21.7-24.1) |
| RV GCS | 16.0 (15.6-16.3) ^†^ | 14.8 (13.9-15.7) |
| RV longitudinal PEDSR | 0.77 (0.74-0.80) ^†^ | 0.93 (0.84-1.0) |
| RV circumferential PEDSR | 0.56 (0.54-0.57) | 0.60 (0.55-0.65) |
| RV longitudinal PLDSR | 1.23 (1.18-1.27) | 1.14 (1.00-1.27) |
| RV circumferential PLDSR | 0.32 (0.31-0.34) ^†^ | 0.26 (0.21-0.31) |
| Echocardiography | | |
| E wave | 70 (68-72) | 69 (65-75) |
| A wave | 81 (80-83) ^†^ | 75 (69-80) |
| E/A ratio | 0.88 (0.85-0.90) ^†^ | 0.96 (0.89-1.0) |
| E/e’ | 9.1 (8.9-9.4) | 8.8 (8.1-9.6) |

Baseline characteristics are presented as either mean±standard deviation, median (interquartile range) or n (%) as appropriate. Imaging variables are presented as adjusted means (95% confidence intervals).

Abbreviations: T2D = Type 2 diabetes, BMI = body mass index, BP = blood pressure, RV = right ventricle, EDVi – indexed end-diastolic volume, ESVi =indexed end-systolic volume, GLS = global longitudinal strain, GCS = global circumferential strain, PEDSR = peak early diastolic strain rate, PLDSR = peak late diastolic strain rate.

*= P<0.05 compared to controls.

^†^ = P<0.05 compared to controls adjusted for age, sex, race and body mass index

|  | Observer 1  Mean ± SD | Observer 2  Mean ± SD | Intraclass correlation coefficient | Bias | LLOA | ULOA |
| --- | --- | --- | --- | --- | --- | --- |
| RV end-diastolic volume (mL) | 177 ± 41 | 170 ± 40 | 0.987 | 6.8 | -6.352 | 19.95 |
| RV end-systolic volume (mL) | 79 ± 19 | 76 ± 19 | 0.956 | 3.533 | -10.79 | 17.86 |
| RV ejection fraction (%) | 55 ± 3 | 56 ± 4 | 0.706 | -0.306 | -7.683 | 7.071 |
| RV mass (g) | 24 ± 6 | 26 ± 6 | 0.936 | -1.831 | -6.607 | 2.945 |

**Supplementary Table S5: Inter-observer variability for key RV parameters**

Abbreviations: RV = right ventricle, SD = standard deviation, LLOA = lower limit of agreement, ULOA = upper limit of agreement

**Supplementary Table S6: CPET data from participants with type 2 diabetes and controls who achieved respiratory exchange ratio ≥1.0.**

|  | T2D  (n=279) | Controls  (n=64) | P value* |
| --- | --- | --- | --- |
| Exercise duration (mins) | 10.5 (10.3-10.7) | 10.4 (9.9-10.8) | 0.582 |
| Peak workload (W) | 123 (119-127) | 161 (153-170) | **<0.001** |
| % Workload achieved | 81(79-84) | 106 (101-111) | **<0.001** |
| Peak VO_2_ (mL/kg/min) | 20.3 (19.8-20.9) | 23.3 (22.2-24.5) | **<0.001** |
| Peak RER | 1.09 (1.08-1.10) | 1.11 (1.09-1.12) | 0.085 |
| Resting O_2_ pulse | 4.0 (3.9-4.2) | 4.8 (4.4-5.2) | **<0.001** |
| Peak O_2_ pulse | 11.5 (11.2-11.8) | 13.1 (12.4-13.8)7 | **<0.001** |
| Peak heart rate (bpm) | 152 (150-154) | 157 (152-161) | 0.066 |

Values presented are adjusted means (95% confidence interval).

Abbreviations: T2D = type 2 diabetes, RER = respiratory exchange ratio.

*Adjusted for age, sex, race and body mass index

**Supplementary Table S7: Correlation of imaging parameters with Peak VO_2_ in type 2 diabetes in those with respiratory exchange ratio ≥ 1.0 (n= 279).**

|  | Correlation co-efficient | P value |
| --- | --- | --- |
| RV EDV | 0.183 | **0.002** |
| RV ESV | 0.160 | **0.008** |
| RV EF | -0.063 | 0.296 |
| RV Cardiac output | 0.211 | **<0.001** |
| RV Mass | 0.143 | **0.018** |
| RV Mass:volume | -0.111 | 0.068 |
| RV GCS | -0.055 | 0.376 |
| RV GLS | 0.082 | 0.177 |
| RV Longitudinal PEDSR | 0.114 | 0.063 |
| RV Circumferential PEDSR | 0.033 | 0.601 |
| RV Longitudinal PLDSR | -0.013 | 0.831 |
| RV Circumferential PLDSR | -0.144 | **0.023** |
| RA maximum area | 0.158 | **0.010** |
| RA EF | -0.094 | 0.125 |
| PA diameter | -0.029 | 0.632 |
|  |  |  |
| LV EDV | 0.108 | 0.072 |
| LV ESV | 0.109 | 0.070 |
| LV EF | -0.073 | 0.228 |
| LV Cardiac output | 0.132 | **0.026** |
| LV Mass | 0.109 | 0.070 |
| LV Mass:volume | -0.026 | 0.666 |
| LV GCS | 0.064 | 0.287 |
| LV GLS | -0.037 | 0.536 |
| LV Longitudinal PEDSR | 0.093 | 0.126 |
| LV Circumferential PEDSR | 0.007 | 0.905 |
| LV Longitudinal PLDSR | -0.032 | 0.602 |
| LV Circumferential PLDSR | 0.006 | 0.918 |
| Extracellular volume fraction | -0.052 | 0.394 |
| LA maximum volume | -0.029 | 0.635 |
| LA EF | 0.171 | **0.005** |
| RV EDV/LV EDV ratio | 0.161 | **0.008** |
| MVPA | 0.025 | 0.716 |
| E/A ratio | 0.076 | 0.207 |
| E/e’ | -0.194 | **0.001** |
| MV deceleration time | 0.081 | 0.184 |

Abbreviations: RV = right ventricle, LV = left ventricle, RA = right atrium, LA = left atrium, PA = pulmonary artery, EDVi = indexed end-diastolic volume, ESVi = indexed end-diastolic volume, EF = ejection fraction, GCS = global circumferential strain, GLS = global longitudinal strain, PEDSR = peak early diastolic strain rate, PLDSR = peak late diastolic strain rate, PCWP = pulmonary capillary wedge pressure.

**Supplementary Table S8: Clinical multivariable linear regression model for predicting weight-adjusted peak VO_2_ in the type 2 diabetes group.**

|  | Unstandardized Coefficients | | Standardized Coefficients | P value |
| --- | --- | --- | --- | --- |
|  | B | Standard Error | Beta |  |
| Age | -0.217 | 0.040 | -0.282 | **<0.001** |
| Male | 4.200 | 0.542 | 0.385 | **<0.001** |
| Non-white | -2.493 | 0.499 | -0.242 | **<0.001** |
| Body mass index | -0.383 | 0.050 | -0.375 | **<0.001** |
| Smoking status | -0.740 | 0.387 | -0.094 | 0.057 |
| Systolic BP | -0.046 | 0.022 | -0.104 | **0.036** |
| Heart rate | -0.066 | 0.030 | -0.119 | **0.027** |
| eGFR | -0.002 | 0.017 | -0.005 | 0.921 |
| HbA1c % | -0.181 | 0.221 | -0.041 | 0.413 |

R=0.665, R square=0.442, Adjusted R square=0.422, P<0.001

Abbreviations: BP = blood pressure.

|  | Unstandardized Coefficients | | Standardized Coefficients | P value |
| --- | --- | --- | --- | --- |
|  | **B** | **Standard Error** | **Beta** |  |
| Age | -0.238 | 0.114 | -0.315 | 0.054 |
| Male | -0.411 | 2.159 | -0.034 | 0.852 |
| Non-white | -3.414 | 2.326 | -0.261 | 0.162 |
| Body mass index | -0.655 | 0.242 | -0.462 | **0.016** |
| Smoking status | -3.929 | 1.673 | -0.340 | **0.032** |
| Ambulatory systolic BP | 0.009 | 0.077 | 0.019 | 0.908 |
| Ambulatory heart rate | -0.154 | 0.139 | -0.169 | 0.284 |
| eGFR | -0.063 | 0.077 | -0.103 | 0.428 |
| HbA1c % | -1.393 | 2.031 | -0.095 | 0.503 |
| E/e’ | -1.020 | 0.557 | -0.265 | 0.086 |
| Myocardial perfusion reserve | 2.656 | 0.879 | 0.372 | **0.008** |
| LA EF | -0.061 | 0.103 | -0.084 | 0.562 |
| RV EDV | 0.120 | 0.048 | 0.848 | **0.024** |
| RV Cardiac output | -1.009 | 0.643 | -0.294 | 0.136 |
| RV Circumferential PLDSR | 9.773 | 8.253 | 0.192 | 0.254 |
| RV EDV/LV EDV ratio | -3.367 | 10.430 | -0.055 | 0.751 |
| RA maximum area | -0.395 | 0.244 | -0.343 | 0.125 |

**Supplementary Table S9: Multivariable linear regression model for predicting weight-adjusted peak VO_2_ in the control group who achieved respiratory exchange ratio ≥ 1.0.**

R=0.917, R square= 0.842, Adjusted R square = 0.673, P=0.001.

Abbreviations: RER = respiratory exchange ratio, BP = blood pressure, LA = left atrial, EF = ejection fraction, RV = right ventricle, EDVi = indexed end diastolic volume, PLDSR = peak late diastolic strain rate, RA = right atrium, LV = left ventricle
